# Long-term, sustainable solutions to radioactive waste management

**DOI:** 10.1038/s41598-024-55911-y

**Published:** 2024-03-11

**Authors:** Kristina Kvashnina, Francis Claret, Nicolas Clavier, Tatiana G. Levitskaia, Haruko Wainwright, Tiankai Yao

**Affiliations:** 1 Institute of Resource Ecology, Helmholtz-Zentrum Dresden-Rossendorf, Bautzner Landstrasse 400, 01328 Dresden, Germany; 2 The Rossendorf Beamline at ESRF, CS 40220, 38043 Grenoble Cedex 9, France; 3 BRGM, 3, Avenue Claude Guillemin, 45060cedex 2 Orléans, France; 4 ICSM, University of Montpellier, CEA, CNRS, ENSCM, Marcoule, France; 5 Pacific Northwest National Laboratory, 902 Battelle Blvd, Richland, WA 99354 USA; 6 Massachusetts Institute of Technology, Cambridge, MA USA; 7 Idaho National Laboratory, Idaho Falls, ID 83415 USA

**Keywords:** Nuclear waste, Geochemistry

## Abstract

Nuclear power plays a pivotal role in ensuring a scalable, affordable, and reliable low-carbon electricity supply. Along with other low-carbon energy technologies, nuclear energy is essential for reducing our reliance on fossil fuels, addressing climate change and air pollution, and achieving a sustainable economy. Whilst significant progress has been made in reducing the volume of final radioactive waste, its management remains one of the most important challenges when considering the continued use and expansion of nuclear energy. This recently published collection highlights the latest technological and scientific advances aimed to improve the safe, long-term, and sustainable management of wastes produced from nuclear power generation.

Nuclear power is an ideal option for sustainable energy generation due to its long operating life and its ability to generate electricity with minimal greenhouse gas emissions. However, this form of energy generation produces radioactive waste that must be either securely stored and disposed or subjected to reprocessing. Long-term, sustainable solutions for radioactive waste management require a combination of technical expertise, regulatory oversight, and ongoing research to ensure the safe containment and ultimate disposal.

One approach is to establish deep geological repositories in stable geological formations. These repositories should be designed to isolate radioactive waste from the environment for thousands of years. In this context, Finkeldei, Huitinnen and colleagues investigated zirconia (ZrO_2_) formed on spent nuclear fuel rod cladding and demonstrated its potential as an engineered barrier for immobilizing radionuclides^1^. They achieved this by structurally incorporating Eu^3+^ and Cm^3+^ into zirconia and analyzed the materials using complementary methods, including powder X-ray diffraction (PXRD), spectrum imaging analysis based on energy-dispersive X-ray spectroscopy in scanning transmission electron microscopy mode (STEM-EDXS), and luminescence spectroscopy. They showed that zirconia could be a suitable technical retention barrier for mobilized trivalent actinides in deep geological repositories.

Ceramic materials are also considered as leading candidate waste forms for the immobilization and geological disposal of long-lived actinides. In this collection, Corkhill et al.^2^ investigated titanate ceramics with the pyrochlore structure using photoemission and X-ray absorption spectroscopy (XAS) and showed the possibility of chemical flexibility in terms of crystal-chemical design principles of pyrochlore structure. Additionally, Corkhill et al.^3^ explored the mineral brannerite UTi_2_O_6_ as a possible host phase for the immobilization of long-lived actinides, such as plutonium. Their investigation demonstrated that the ThTi_2_O_6_ brannerite structure can incorporate a small fraction of U^6+^ alongside a more significant inventory of U^5+^, achieved through charge compensation on the Th or Ti site. Blackburn et al.^4^ conducted research on zirconolite as a candidate waste form material for immobilizing actinides from spent nuclear fuel. They considered indium as a neutron-absorbing additive to mitigate criticality in ceramic waste forms. The recorded K-edge spectroscopy data on In and Zr provide valuable information about the coordination environment and oxidation states of In and Zr, contributing to the performance testing of titanate waste form materials. Anionic clays like hydrotalcites are being explored, but their ability to serve as a durable repository for long-term actinide isolation is uncertain. Douglas and co-authors^5^ showed that nanoscale hydrotalcite efficiently captured radionuclides, suggesting a potential rapid decontamination and long-term containment solution. Finally, spent nuclear fuel itself can be considered as a suitable wasteform, leading to its direct storage in geological repositories. In this context, Vinograd and co-workers^6^ questioned the reactivity of Ln-doped UO_2_ samples, as model compounds for SNF, towards oxidation in air. They showed that the partitioning of lanthanide cations between the original fluorite-type phase and the oxidized U_3_O_8_ enhanced the resistivity to oxidation, thus contributing to the chemical durability of the ceramic.

Investing in fundamental research and the development of new technologies for waste treatment and disposal can lead to more efficient and cost-effective methods. Our collection features several articles on fundamental science, with a particular focus on investigating the electronic structure of actinide materials, which plays a significant role in radioactive waste management. Butorin and his co-authors^7^ conducted research on americium oxide, an important component of the nuclear fuel cycle, using soft XAS and electronic structure calculations. Their study revealed that AmO_2_ can be classified as a charge-transfer compound with a 5f. occupancy of 5.73 electrons, while Am_2_O_3_ exhibits characteristics of a Mott–Hubbard system with a 5f occupancy of 6.05.

Investigating nuclear accidents is crucial for improving nuclear waste solutions because it enhances safety, reduces risks, strengthens regulations, drives technological advancements, and encourages international cooperation to address the challenges of nuclear waste management. Poliakova et al.^8^ studied uranium oxides, which can disperse into the environment in various forms following different accidental scenarios. The team investigated the behavior of uranium oxide particles, which could be easily ingested by humans and animals in the vicinity of a contaminated area. Using several experimental methods, they observed structural changes in mixed uranium oxides, specifically UO_2,_ U_4_O_9_, U_3_O_8_ and UO_3_, before and after exposure in simulated biological fluids (gastro-intestinal and lung). The most significant changes were observed in U_4_O_9_. This study heavily utilized XAS techniques at synchrotron sources, highlighting the crucial role of synchrotron radiation in nuclear waste research.

Understanding the chemical forms, defects, crystal structures, and other properties of radioactive materials is crucial before the construction of a final geological repository. While underpinning radionuclides speciation is a key asset to dive its mobility, reactive transport code allow to model their behavior on a long term scale. Tournassat et al.^9^ have developed a user-friendly numerical reactive transport approach in order to tackle diffusion experiment in a smarter way. Reactive transport is a powerful tools but when uncertainty analysis have to be conducted, the computing cost could be a limiting factor. To overcome this issue surrogate model can be used. Turunen and Lipping^10^ have built surrogate models using convolutional neural networks and used the meta model for a sensitivity analysis of the radionuclide transport models. In a separate work, Butorin and the team^11^ investigated the effect of carbon content on the electronic structure of uranium carbides through a combination of XAS and density functional theory that takes 5f–5f Coulomb interaction U and spin–orbit coupling (DFT + U + SOC) into consideration. The results revealed an increase amount of 5f electrons in UC_2_ as compared to UC, which will impact the thermodynamic properties and eventually on the carbide nuclear fuel performance.

Transmutation of radionuclides have been considered as one of the potential solutions for managing high-level radioactive waste. Sun et al.^12^ proposed a new framework to transmute not only minor actinides but also long-lived fission products based on lead-cooled fact reactors. Using OpenMC, the optimal MA loading is evaluated based on the operation of the core, the neutron fux distribution, spectra, and k_*eff*_. They have shown the premise of transmuting mobile and key radionuclides for geological disposal such as Tc-99, with the higher transmutation rate than 15%. One more aspect, as proper decommissioning of nuclear facilities and reactors, followed by the management of resulting waste, is a critical component of long-term solutions. In this field, different strategies can be envisaged to lower both the volume and the radiotoxicity of the waste. The first one lies on the reprocessing of spent fuel in order to separate the different isotopes to offer targeted solutions for their storage. In this frame, Pilgrim et al.^13^ proposed novel aminocarboxylate chelators providing highly efficient complexation of trivalent lanthanides and minor actinides. They particularly studied structural modifications of the ligands that increased their solubility without influencing their ability to complex lanthanides and minor actinides in solution.

Overall, we believe there are many key aspects which play a role in finding long-term solutions for radioactive waste. Proper classification of radioactive waste based on its characteristics and levels of radioactivity is essential. Strict safety standards and regulations are crucial to safeguard workers, the public, and the environment. Radioactive waste management often involves international cooperation, as some countries may not have the resources or expertise to manage their waste independently. Our collection reflects the international collaboration between scientists in different fields and in world-wide locations.
